# Genetic Marker Suitable for Identification and Genotyping of Plasmodium ovale curtisi and Plasmodium ovale wallikeri

**DOI:** 10.1128/JCM.01527-13

**Published:** 2013-12

**Authors:** Naowarat Tanomsing, Mallika Imwong, Colin J. Sutherland, Christiane Dolecek, Tran Tinh Hien, Francois Nosten, Nicholas P. J. Day, Nicholas J. White, Georges Snounou

**Affiliations:** Department of Molecular Tropical Medicine and Genetics, Faculty of Tropical Medicine, Mahidol University, Bangkok, Thailanda; Immunology Unit, Department of Infectious and Tropical Diseases, London School of Hygiene and Tropical Medicine, London, United Kingdomb; Hospital for Tropical Diseases, London, United Kingdomc; Oxford University Clinical Research Unit, Hospital for Tropical Diseases, Ho Chi Minh City, Vietnamd; Centre for Tropical Medicine, Nuffield Department of Clinical Medicine, Churchill Hospital, Oxford, United Kingdome; Shoklo Malaria Research Unit, Mae Sot, Thailandf; Mahidol-Oxford Tropical Medicine Research Unit, Faculty of Tropical Medicine, Mahidol University, Bangkok, Thailandg; Institut National de la Santé et de la Recherche Médicale, Unité Mixte de Recherche S945, Paris, Franceh; Université Pierre and Marie Curie, Faculté de Médecine Pitié-Salpêtrière, Paris, Francei

## Abstract

We present a seminested PCR method that specifically discriminates between Plasmodium ovale curtisi and P. ovale wallikeri with high sensitivity. The test is based on species-specific amplification of a size-polymorphic fragment of the tryptophan-rich antigen gene, *potra*, which also permits discrimination of intraspecific sequence variants at this locus.

## TEXT

Plasmodium ovale has long been considered a parasite that is predominantly found in Tropical Africa, most often in West Africa, and in some islands in the Western Pacific (1). Confirmed cases have occasionally been reported from other regions in which the parasite is endemic, except for the Americas (2, 3). Four factors have contributed to the perception that this species is relatively rare. The clinical course is short and comparatively mild (there are very few records of severity or mortality), the parasite burdens are generally low (peak parasitemias rarely exceed 25,000 parasites per μl of blood in naive individuals), the species is often misdiagnosed in areas where P. ovale is not known to be endemic, and finally, accurate diagnosis by microscopy examination, especially of thick smears, is difficult (2). The last two obstacles were circumvented by the introduction of sensitive molecular techniques (4). These provided the first indication that the prevalence and geographic range of P. ovale were likely to have been underestimated (5). Molecular-based detection also revealed a dimorphism in the P. ovale A-type small subunit rRNA (ssrRNA) genes (6, 7), which extended to other genes (8). Multilocus sequence analysis of isolates from diverse geographical origins culminated in the proposal that there were actually two species, P. ovale curtisi (classic type) and P. ovale wallikeri (variant type) (9). These two species are globally distributed and sympatric (6, 9–14).

In the context of a long-term goal to achieve malaria elimination, it becomes important to understand the epidemiology of P. ovale, a species which is more widespread than previously understood and which shares with P. vivax the formation of hypnozoites that cause relapses (15, 16). Recent observations suggest that the species might differ in their relapse patterns (17). Given the generally low parasite burdens, future investigations must incorporate molecular methods for sensitive detection and identification of the two species, as well as a means to discriminate between different strains using polymorphic markers. A number of protocols based on the ssrRNA genes (10, 12, 18) are suitable for identification but not for genotyping. Sequence and size variations were noted between the tryptophan-rich antigen genes (*poctra* and *powtra*) from P. ovale curtisi and P. ovale wallikeri (9). This was exploited in a nested PCR detection assay (11), where primers target sequences conserved between these two genes and the species are discriminated by the size of the amplified fragments (299 bp or 317 bp for *poctra*; 245 bp for *powtra*). The amplified fragment size variations result from differences in the number of repeated units, which suggests that a broader spectrum of size variants, possibly overlapping for P. ovale curtisi and P. ovale wallikeri, respectively, might occur. This would invalidate amplified fragment size difference as a means of distinguishing between P. ovale curtisi and P. ovale wallikeri.

**Fig 1:**
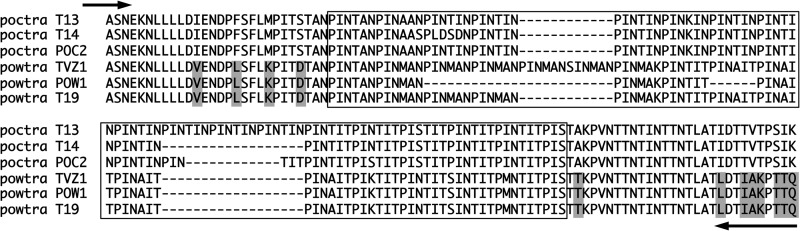
Predicted amino acid alignment of the distinct *potra* fragments amplified from P. ovale curtisi and P. ovale wallikeri. Boxed sequences represent the repetitive regions that were seen to vary in length between isolates. Shaded residues indicate those that appear to be specific to P. ovale wallikeri genes. Black arrows indicate the positions of the oligonucleotides used in the secondary amplification reaction to generate the size variants observed for *poctra* and *powtra*: at the 5′ end, the PoTRA-F primer whose target includes the 12 bp preceding the open reading frame recognizes the gene from both species, while two 3′-end primers were designed to specifically recognize the *poctra* or *powtra* gene.

When a set of P. ovale isolates collected from Thailand (*n* = 9; T series) and Vietnam (*n* = 2; V series) were tested using the species-conserved *potra* oligonucleotides (11), a broader range of fragment sizes than that noted previously (11) was observed, with some overlap between the two species (Table 1). Consequently, we designed a new set of primers suitable for species-specific seminested PCR. The oligonucleotide primers were designed based on the *potra* gene sequences available in GenBank (accession no. HM594182 to HM594183 for P. ovale curtisi, accession no. HM594180 to HM594181 for P. ovale wallikeri). For the primary reaction, a fragment of ca. 705 bp spanning the repeat region of *poctra* and *powtra* was amplified using oligonucleotides targeting regions conserved between the two species by using a new primer, PoTRA-F (5′-CATTTTACGTAGGCATCTAA-3′), which targets the 5′ end of the gene, and the previously published PoTRA rev3 (11). For the secondary amplification reaction, PoTRA-F was used in two separate reactions, but in this case with an oligonucleotide specific to each of the two species, either PocTRA-R (5′-TTTATGGATGGTGTGACTGTTGTATCTATA-3′) or PowTRA-R (5′-TGTGTGGTTGGTTTGACTATCGTATCTAAG-3′) was used for P. ovale curtisi and P. ovale wallikeri, respectively (Fig. 1). The amplification conditions for the primary reaction were optimized by using genomic DNA isolated from P. ovale curtisi (T13)- or P. ovale wallikeri (TVZ1)-infected blood samples (with respect to annealing temperature as well as Mg^2+^ and oligonucleotide concentrations). The fragments obtained for each species were then cloned into the pCR 2.1 vector (Invitrogen, USA), and each plasmid was purified from the bacterial clones. These standard plasmids were used to optimize the conditions for the secondary amplification reactions (with respect to annealing temperature as well as Mg^2+^ and oligonucleotide concentrations) and to derive the limit of detection of the seminested PCR protocol. The concentration of each standard plasmid stock solution was determined by using the optical density of the solution at 260 nm. The copy number of each standard plasmid per μl was calculated as the mass of the plasmid standard (g/μl) divided by the calculated mass of each molecule (number of bp × 660 g/6.027 × 10^23^). A serial dilution series, in which there were 1, 2, 5, 10, 10^2^, 10^3^, 10^4^, or 10^5^ copies per μl, was then obtained, and 1 μl of each dilution was tested five times.

**Table 1:** Sizes of the sequenced *potra* fragments amplified using the different primer pairs

Sample(s)	P. ovale subspecies	Potra fwd5 + Potra rev5 (bp)	PoTRA-F + PocTRA-R (bp)	PoTRA-F + PowTRA-R (bp)
POW1 or POW2^*a*^	wallikeri	245		389
11 (Africa)	wallikeri	245		389
T7, T9, T11, T19^*b*^	wallikeri	299		443
T22 (+P. vivax)	wallikeri	299		443
T12 (+P. falciparum)	wallikeri	299		443
VP, TVZ1^*c*^	wallikeri	335		479
POC1^*d*^	curtisi	299	443	
VN, T14^*e*^	curtisi	299	443	
POC2^*d*^	curtisi	317	461	
T13^*f*^	curtisi	353	497	

a POW1 and POW2 sequences were previously obtained (GenBank accession no. HM594180 and HM594181, respectively).

b GenBank accession no. for T19 is KF018430.

c GenBank accession no. for TVZ1 is KF018431.

d POC1 and POC2 sequences were previously obtained (GenBank accession no. HM594182 and HM594183, respectively).

e GenBank accession no. for T14 is KF018433.

f GenBank accession no. for T13 is KF018432.

All reactions were carried out in a total volume of 20 μl in the presence of 10 mM Tris-HCl (pH 8.3), 50 mM KCl, 75 or 125 μM for each deoxyribonucleotide triphosphate, and 0.4 units of *Taq* polymerase (Invitrogen, USA). The primary amplification was carried out at a final concentration of 2 mM MgCl_2_ and 75 nM for each primer, with an annealing temperature of 56°C, whereas the secondary amplification was carried out using an annealing temperature of 60°C, with a final concentration of 3 mM for MgCl_2_ and 125 nM for each primer. One microliter of template was used to initiate both the primary and secondary amplification reactions. The cycling parameters consisted of an initial denaturation step at 95°C for 5 min, annealing for 1 min, and then extension at 72°C for 1 min, followed by a denaturation step at 94°C for 1 min. After a given number of cycles (25 cycles for the primary amplification and 30 cycles for the secondary amplification), a final extension step at 72°C was carried out before storage of the product at 4°C. Ten microliters of the secondary reaction products was electrophoresed on a 2% agarose gel, and the bands were then visualized by UV transillumination, following staining with ethidium bromide.

The limit of detection, as based on the template with a known number of plasmid molecules, was five copies, for which all five duplicates gave a positive result. The specificity of the reaction was confirmed by using high concentrations of genomic DNA (equivalent to 10^4^ parasite genomes) from P. falciparum, P. vivax, P. malariae, or human DNA as templates alone (all reactions proved negative) or mixed with one or other of the standard plasmid templates that demonstrated that sensitivity was not affected. No detectable fragments could be amplified when plasmid DNA carrying the *potra* fragment from one P. ovale species was used as a template for the secondary amplification reaction, in which the primer pair specific for the *potra* of the other P. ovale species was used. The sensitivity and specificity of the protocol was then assessed by using genomic DNA purified from clinical blood samples containing P. ovale curtisi and P. ovale wallikeri that had been enumerated accurately (416 parasites/μl blood and 1,152 parasites/μl blood). These genomic DNAs were then serially diluted and assayed. The seminested protocol was able to consistently detect a parasitemia equivalent to 2 to 10 parasites/μl blood. The specificity and the consistency of the sensitivity were again confirmed by adding excess P. falciparum, P. vivax, P. malariae, or human genomic DNAs to the serially diluted DNA. Finally, genomic DNA templates from 30 patients infected with P. falciparum (*n* = 10), P. vivax (*n* = 10), or P. malariae (*n* = 10) were also tested and proved negative.

**Fig 2:**
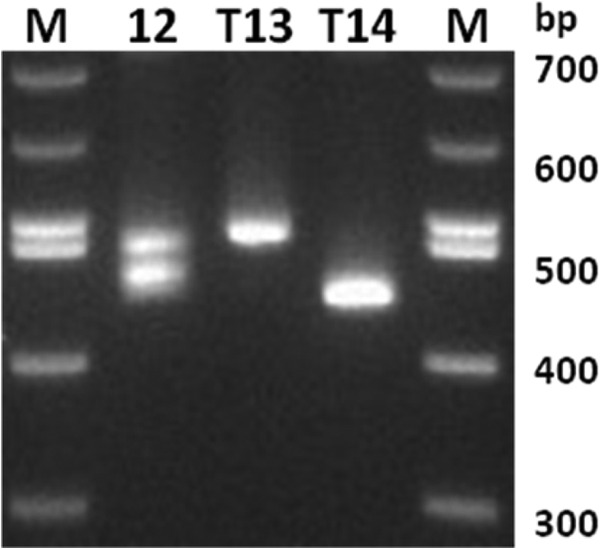
Amplified *poctra* fragments from three P. ovale curtisi isolates. Sample 12 (of African origin) had a mixed-genotype infection; T13 and T14 contain an amplified fragment of 497 bp and 443 bp. Lane M represents the 100 bp molecular weight marker (the 500 bp is the lower band).

The seminested PCR protocol was then applied to DNA purified from 17 clinical blood samples: 7 samples infected with P. ovale curtisi (two of these were mixed infections with P. falciparum) and 10 samples infected with P. ovale wallikeri (two of these were mixed infections, one with P. falciparum and the other with P. vivax). The species present in these samples had been previously established by analysis of the ssrRNA genes and the mitochondrial locus *pocytb* (9). The *potra*-based protocol presented here correctly identified the species present in each sample. Moreover, the isolates from each species could be classed according to the amplified fragment size. Three distinct allelic *potra* variants were sequenced for each species, and the predicted amino acid sequences were aligned (Fig. 1). Subsequent to this, two potentially new size variants were amplified (Fig. 2) from a P. ovale curtisi sample recently collected from a patient who had acquired the infection in Africa (the exact country had not been recorded).

Thus, we present a sensitive seminested PCR protocol that not only allows discrimination between P. ovale curtisi and P. ovale wallikeri with high specificity but also provides a simple means to identify genotypic variants within each of these species. We are aware that nested PCR protocols are associated with two disadvantages. The first is the additional cost and labor of carrying out an additional PCR. The second is the substantially increased risk of contamination inherent to the transfer of the PCR product from the first to the second reaction; in our experience, this risk can be substantially reduced by the allocation of a distinct laboratory space for this transfer. We feel that in this particular case, the use of a seminested PCR protocol can be justified. The limit of detection of nested PCR-based protocols is often higher than that of methods based on a single amplification step, an important consideration for P. ovale infections where parasite burdens are often quite low. Moreover, the nested PCR format is less sensitive to inhibitors present in the initial template. Finally, discrimination of allelic variants by size is most practically carried out following gel electrophoresis, a step that will negate any advantage of methods where the amplified product remains in closed tubes. Ultimately, the protocol presented here is intended for fundamental investigations on the two P. ovale species and not for implementation in a routine laboratory, as there is no evidence that the clinical course or the treatment required varies between P. ovale wallikeri and P. ovale curtisi infections.

As more samples are analyzed, it is likely that the number of *potra* size variants that occur would exceed those observed to date (five for *poctra* and three for *powtra*). In conclusion, the *potra* genes could now serve as targets for molecular identification and as genetic markers suitable for a broad range of investigations of the epidemiology and biology of P. ovale curtisi and P. ovale wallikeri, similar to those carried out for P. falciparum and P. vivax (19–21).

### Nucleotide sequence accession numbers.

The *potra* gene sequences for samples T19, TVZ1, T13, and T14 were deposited in GenBank under accession numbers KF018430 to KF018433, respectively.
